# Translational Fidelity during Bacterial Stresses and Host Interactions

**DOI:** 10.3390/pathogens12030383

**Published:** 2023-02-28

**Authors:** Zhihui Lyu, Cierra Wilson, Jiqiang Ling

**Affiliations:** Department of Cell Biology and Molecular Genetics, The University of Maryland, College Park, MD 20742, USA

**Keywords:** bacterial infections, mistranslation, aminoacyl-tRNA synthetases, ribosome

## Abstract

Translational fidelity refers to accuracy during protein synthesis and is maintained in all three domains of life. Translational errors occur at base levels during normal conditions and may rise due to mutations or stress conditions. In this article, we review our current understanding of how translational fidelity is perturbed by various environmental stresses that bacterial pathogens encounter during host interactions. We discuss how oxidative stress, metabolic stresses, and antibiotics affect various types of translational errors and the resulting effects on stress adaption and fitness. We also discuss the roles of translational fidelity during pathogen–host interactions and the underlying mechanisms. Many of the studies covered in this review will be based on work with *Salmonella enterica* and *Escherichia coli*, but other bacterial pathogens will also be discussed.

## 1. Introduction

Protein synthesis is a multistep and extensively regulated process central to all cells. It is estimated that 70% of cellular ATP is consumed to synthesize proteins [1]. The ribosome makes proteins using mRNAs as the template and aminoacyl-tRNAs (aa-tRNAs) as substrates [2,3]. The correct pairing of mRNA codons and tRNA anticodons ensures that the genetic information stored in DNA (and passed to mRNAs) is accurately reflected in the protein sequence. It is well established that both the initial selection of cognate aa-tRNAs and subsequent kinetic proofreading against near-cognate aa-tRNAs are critical for maintaining decoding fidelity on the ribosome [4,5]. Another important step to ensure translational fidelity is aa-tRNA synthesis, during which amino acids are attached to the corresponding tRNAs by specialized aminoacyl-tRNA synthetases (aaRSs) [6]. Due to the structural similarity between different amino acids, the active site of aaRSs often fails to adequately distinguish between the correct and incorrect amino acids; many aaRSs thus use pre- or posttransfer editing to proofread the aa-tRNAs and prevent the accumulation of misacylated tRNAs [7,8]. In addition, free-standing editing factors provide another sieve to remove misacylated tRNAs in trans [9,10]. Collectively, these quality control mechanisms lead to a base-level amino acid misincorporation rate of ~1 in 10,000 decoding events (reviewed in [11]). Such error rates result in approximately 10% of the proteins containing at least one amino acid misincorporation, a level well tolerated by cells [12]. Mutations in translational factors and aminoglycoside antibiotics may increase missense errors to 10^−3^–10^−2^ [11,13,14]. Compared with missense errors, stop-codon readthrough occurs at a higher frequency of 10^−3^ to 10^−2^ [15,16,17,18,19,20]. Mutations and environmental stresses may further increase readthrough errors to ~10% [15]. In this review, we discuss the genetic and environmental factors that affect bacterial translational fidelity in the context of host-related stress conditions as well as how changing translational fidelity affects bacterial interaction with the host.

## 2. Translational Fidelity during Bacterial Stresses

**Figure 1:**
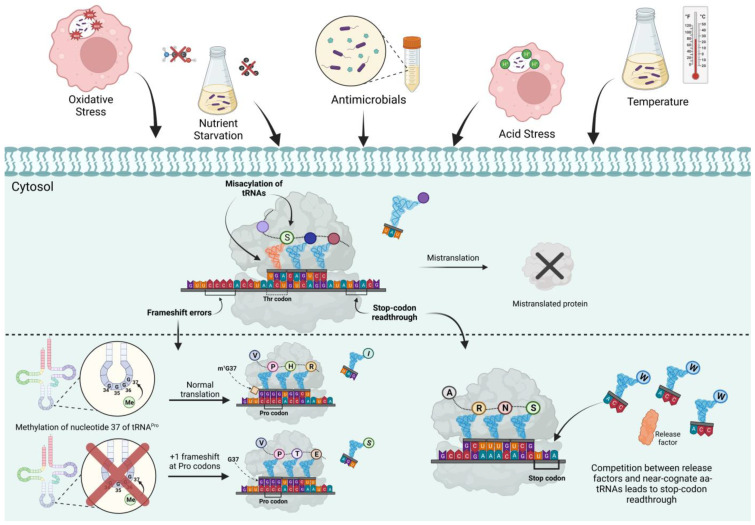
Translational fidelity is altered by environmental cues. Translational fidelity is maintained through the correct attachment of amino acids to tRNAs and accurate decoding on the ribosome. Environmental cues, such as oxidative stress, nutrient starvation, acid stress, and antimicrobials, have been shown to alter translational fidelity, which results in various changes in bacterial fitness and host interactions.

Bacteria are frequently exposed to stressful conditions such as oxidants, heat, nutrient starvation, acids, and antibiotics [21,22]. Many of the stresses are experienced by pathogens during host infections [23]. For instance, bacterial infections activate macrophages and neutrophils to produce reactive oxygen and nitrogen species, and acidic pH is found in the gastrointestinal and genital tracts and intracellular phagolysosomes [23,24,25]. This section reviews how different stresses affect translational fidelity and how translational errors influence bacterial stress resistance (Figure 1 and Table 1).

### 2.1. Effects of Oxidative Stress on Translational Fidelity

Reactive oxygen species (ROS) such as superoxide and hydrogen peroxide (H_2_O_2_) are produced by phagocytes and also as by-products during bacterial respiration [23,54]. H_2_O_2_ reacts with iron to generate highly reactive hydroxyl radicals (OH·) [55]. ROS can oxidize various amino acid residues including cysteine and methionine. The editing sites of threonyl- (ThrRS) and alanyl- (AlaRS) tRNA synthetases both contain a cysteine that is critical for editing misacylated Ser-tRNAs [56,57,58]. An earlier report shows that the editing site cysteine of *E. coli* ThrRS (C182) is susceptible to oxidation by H_2_O_2_ [31]. ThrRS misacylates Ser to tRNA^Thr^ and requires efficient editing to hydrolyze Ser-tRNA^Thr^. Oxidation of ThrRS leads to the accumulation of Ser-tRNA^Thr^ in vitro and Ser misincorporation at Thr codons in vivo, as demonstrated by enzymatic, reporter, and mass spectrometry assays [31]. The following work reveals that ThrRS C182 is oxidized to a sulfenic acid at low micromolar concentrations of H_2_O_2_ [32]. Such sensitivity requires deprotonation of the C182 thiol group by surrounding His residues. Oxidation of ThrRS appears to be well tolerated by wild-type *E. coli* but causes a severe growth defect in the absence of heat-shock proteases [31]. This is in line with studies showing that the ThrRS C182A mutation results in little growth defect [13,48]. In contrast, mutating the editing site Cys (C666 in *E. coli* and C719 in *Saccharomyces cerevisiae*) of AlaRS inhibits growth at elevated temperatures [48,59]. The striking difference between ThrRS and AlaRS editing defects is presumably due to the nature of translational errors. Whereas Ala → Ser replacements increase protein hydrophilicity and destabilize the proteome by increasing protein misfolding and degradation, Thr → Ser changes may be better tolerated due to the similar properties of Thr and Ser. Intriguingly, a recent study revealed that oxidation of *E. coli* AlaRS does not lead to an editing defect, despite oxidation of C666 being detected by mass spectrometry [60]. It is possible that the oxidized form of AlaRS still preserves editing efficiency; alternatively, oxidation of AlaRS may not be complete under tested conditions, and the remaining nonoxidized AlaRS hydrolyzes Ser-tRNA^Ala^ *in trans*. It seems that the AlaRS editing site has evolved to resist oxidative stress and avoid detrimental Ala → Ser misincorporation in the proteome.

Phenylalanyl-tRNA synthetase (PheRS) uses the editing site to hydrolyze misacylated *p*-Tyr-tRNA^Phe^ and prevent misincorporation of *p*-Tyr at Phe codons [61,62,63]. Under oxidative stress, Phe is oxidized to *m*-Tyr, which is a better substrate for PheRS aminoacylation than *p*-Tyr and thus poses a dangerous threat to quality control [64]. Recent work demonstrates that *Salmonella enterica* serovar *typhimurium* PheRS improves editing efficiency under oxidative stress to defend against the toxicity of *m*-Tyr and *p*-Tyr [34]. Oxidation of PheRS occurs at multiple residues, as revealed by mass spectrometry. Cryo-electron microscopy structures of nonoxidized and oxidized PheRS show that oxidation enlarges the editing pocket, which may explain the enhanced editing activity of PheRS upon oxidation [65]. The seemingly opposite effects of oxidation on ThrRS and PheRS editing efficiency are likely linked to the different severity of mistranslation events. As discussed above, Thr → Ser misincorporation resulting from ThrRS editing deficiency is well tolerated, whereas mistranslation of Phe codons with *m*-Tyr and *p*-Tyr impairs fitness under oxidative stress conditions [34,64].

In addition to aaRSs, ribosomal RNAs and proteins are also targets of ROS [66,67]. Oxidation of rRNA impairs various steps of translational elongation [66], and ribosomal proteins undergo reversible or irreversible oxidation under stress conditions [67]. Oxidative stress induced by menadione appears to increase the rates of stop-codon readthrough and frameshift errors in *Staphylococcus aureus*, as shown by dual-luciferase reporters [68]. The underlying mechanism is unclear, and whether oxidative stress affects ribosomal fidelity in other bacteria remains to be determined.

### 2.2. Effects of Metabolic Stresses on Translational Fidelity

Cellular metabolism is heavily influenced by environmental conditions such as nutrient availability, oxygen levels, and pH. Growing evidence suggests that dysregulation of cellular metabolism leads to altered translational fidelity. An earlier study shows that carbon starvation promotes stop-codon readthrough in *E. coli*, although the mechanism remains unclear [30]. Another study reveals that anaerobic and sublethal concentrations of chloramphenicol lower the level of succinyl-CoA, which modifies methionyl-tRNA synthetase at several lysine residues [41]. Decreased MetRS succinylation enhances misacylation of Met to noncognate tRNAs and presumably increases Met misincorporation at non-Met codons [41].

Translational termination at stop codons is mediated by release factors [69]. Kinetic experiments in vitro demonstrate that the activity of release factors decreases under acidic conditions [70,71,72]. We have recently shown that acidic pH caused by an overflow of glucose metabolism promotes stop-codon readthrough, supporting that low pH impairs the release factor activity in vivo [15].

The connection between metabolism and translational fidelity is further revealed by a recent genetic screening [73]. In a genome-wide screening of an *E. coli* knockout library, we have identified several genes that control metabolic processes to affect stop-codon readthrough. In particular, CyaA controls the synthesis of cyclic AMP, which is a master regulator of metabolic pathways [74]. We show that deleting *cyaA* decreases readthrough of stop codons, at least partially by repressing the expression of tRNAs that compete with release factors [73]. It is possible that amino acid imbalance may also contribute to the efficiency of readthrough.

### 2.3. Antibiotics Affecting Translational Fidelity

Aminoglycosides are among the first antibiotics isolated from microbes and used clinically [75]. Aminoglycosides bind the A site of the 30S ribosomal subunit and promote misreading of mRNA codons [35,76]. Ribosomal mistranslation results from stabilization of near-cognate codon–anticodon interactions upon binding of aminoglycosides [77]. Aminoglycoside antibiotics are bactericidal, and the killing effect is thought to be caused by protein mistranslation and misfolding [37,78]. Bacteriostatic antibiotics targeting the ribosome are normally not considered error-inducing, but it is shown that chloramphenicol and spectinomycin indeed promote stop-codon readthrough [16]. How these antibiotics enhance readthrough is not fully understood. It is likely caused by feedback regulation of tRNA expression: slowing ribosome translation enhances expression of rRNAs and tRNAs, which competes with release factors to suppress stop codons [16].

### 2.4. Translational Fidelity and Stress Resistance

Reduced translational fidelity is mostly detrimental to cells by increasing protein misfolding and destabilizing the proteome. However, several studies have shown that certain types of translational errors may be beneficial under certain stress conditions. In *E. coli*, RpoS is a master regulator of the general stress response [21]. Increased translational errors (misincorporation, stop-codon readthrough, and frameshift) caused by *ram* mutations in the ribosomal gene *rpsD* enhance the protein level of RpoS and protect cells against oxidative stress [28,79]. RpoS expression is regulated at transcriptional, translational, and posttranslational levels [21]. It is shown that ribosomal errors lead to a DsrA-dependent increase in RpoS translation. Mistranslated proteins also bind and titrate ClpXP away from degrading RpoS. For an unknown mechanism, RpoS appears to regulate the protein level of RpoH, a sigma factor that controls the expression of heat-shock genes [29]. Ribosomal errors increase the protein level of RpoH in a manner dependent on RpoS, leading to protection of *E. coli* cells under heat stress [29]. In addition to ribosomal mistranslation, increased errors in translation initiation and misincorporation of amino acid analogs also protect *E. coli* against heat [38]. The same types of mistranslation further elevate the SOS response and increase survival in the presence of DNA-damaging antibiotics (e.g., ciprofloxacin) [38]. It is likely that not all translational errors elicit the same stress responses, and there is a fine line between mistranslation-induced stress protection and toxicity. Whether mistranslation and certain types of translational errors affect stress responses in other bacteria remains an interesting question for exploration in future studies. For example, with recent advances in genome engineering, it would be intriguing to systematically engineer Gram-negative and Gram-positive pathogens and determine how increasing and decreasing aminoacylation and ribosomal errors affect resistance to oxidative, heat, and metabolic stresses. Advancement in quantitative proteomics is also necessary to determine changes in the rates of various translational errors under stress and host conditions.

## 3. Altered Translational Fidelity in *Salmonella* and Other Bacteria

### 3.1. Ribosomal Fidelity Mutations in Salmonella

Mutations in ribosomal small subunit protein S12 (uS12, encoded by *rpsL*) have been found to increase translational fidelity and confer resistance to streptomycin [27,80], whereas mutations in uS4 (encoded by *rpsD*) often lead to reduced translational fidelity [27,81]. Given the opposite effect of *rpsL* and *rpsD* mutations on translational fidelity, it is surprising to find that both *rpsL* K42N (high-fidelity) and *rpsD* I199N (error-prone) *Salmonella* mutants have severe defects in the expression of virulence genes, such as those in the SPI1 Type 3 Secretion System and flagellar motility [26]. Such mutations also impair the infection of host cells and colonization in a zebrafish model [26]. Attenuation of SPI1 gene expression in ribosomal mutants is due to the enhanced degradation of the master regulator HilD by the heat-shock protease Lon. It is proposed that increased translational errors in the *rpsD* I199N mutant activate the expression of Lon, whereas *rpsL* K42 mutation decreases intrinsic misfolded proteins, leading to more Lon protease available to degrade HilD [26]. These findings suggest that *Salmonella* has evolved an optimal translational fidelity suited for host invasion. The *rpsL* K42N mutant also shows improved fitness under bile salt stress, which depends on maintaining a high level of intracellular ATP [82].

### 3.2. Modification Defects of tRNAs in Bacterial Pathogens

Transfer RNAs are heavily modified molecules, and modifications in the anticodon loop often perturb the accuracy of ribosomal decoding [83,84]. The mS2i6A37 modification is catalyzed by MiaABC and promotes stop-codon readthrough by near-cognate tRNAs [73,85,86]. In *Salmonella*, deletion of *miaA* induces pleiotropic effects on cell physiology, including decreased growth, altered sensitivity to several amino acids analogs, and hypersensitivity to oxidative and heat stresses [87,88]. MiaA is required for the efficient expression of virulence genes controlled by VirF in *Shigella flexneri* [44] and is also crucial for the virulence of ExPEC in mice [45]. Deleting *miaA* impairs gut colonization, urinary tract infections, and bloodstream infections caused by ExPEC. The level of MiaA changes during stress conditions (e.g., high salt). Both ablation and overproduction of MiaA increase frameshift errors [45]. Whether altering translational fidelity is sufficient to affect the virulence of *Shigella* and ExPEC remains to be determined.

TrmD catalyzes the methylation of guanine at position 37 to form 1-methylguanosine (m^1^G37) of all three tRNA^Pro^ isoacceptors [89,90]. Lack of m^1^G37 results in elevated frequencies of ribosomal +1 frameshift at Pro codons in *E. coli* and *Salmonella* [89,91,92]. M^1^G37 deficiency causes the accumulation of uncharged tRNA and global ribosome stalling, resulting in activation of the stringent response [93]. TrmD is critical for cell growth and is believed to be essential in several bacterial species, including *E. coli*, *Salmonella*, *Bacillus subtilis, Pseudomonas aeruginosa*, and *Streptococcus pneumoniae* [89,94,95,96,97]. The various mutations in the trmD gene severely reduce colony size and impair the growth of *S. typhimurium* [97]. Decreases in m^1^G37 levels in *E. coli* and *Salmonella* cause membrane damage and lower efflux activity, thus sensitizing these bacteria to various classes of antibiotics such as polymyxin B, ampicillin, gentamicin, and rifampicin [98]. *Salmonella*, as an intracellular pathogen, requires Mg^2+^ transport into cells for survival and virulence [99]. At low Mg^2+^ concentration, a decrease in TrmD activity slows down the translation of the Pro-codon-rich leader sequence *mgtL*, which in turn activates transcription of the Mg^2+^ transporter *mgtA* [100].

MnmE and GidA bind together and form a heterodimeric complex to catalyze the addition of a carboxymethylaminomethyl (cmnm) group at the five positions of the tRNA wobble uridine ((c) mnm^5^s^2^U34) [101,102,103]. The absence of this modification results in an increased level of ribosomal frameshift in *E. coli* and *Salmonella* [39,104,105,106]. GidA potentially regulates several cell division genes and proteins; thus, deletion of *gidA* results in a filamentous morphology due to a defect in chromosome segregation [107]. GidA and MnmE impair *Salmonella* growth and play a role in the regulation of virulence, including invasion of intestinal epithelial cells and motility [40]. GidB, which is in the same operon as GidA, is a methyltransferase responsible for N^7^ methylation of G527 (m^7^G572) of the 16S ribosomal RNA [108]. GidB promotes UGA readthrough in *E. coli* [73]. Under nalidixic acid stress, the *gidB* deletion *Salmonella* mutant exhibits reduced motility, filamentous morphology, and smaller colony size compared to the WT [109].

### 3.3. Glu and Asp Misincorporation in Mycobacteria

In many bacteria (e.g., *Mycobacteria*), GatCAB is responsible for converting Glu-tRNA^Gln^ and Asp-tRNA^Asn^ to Gln-tRNA^Gln^ and Asn-tRNA^Asn^, respectively [42,110,111]. GatCAB mutations in *Mycobacterium smegmatis* are found to increase Gln → Glu and Asn → Asp mistranslation [42]. Intriguingly, such mistranslation events lead to the production of RNA polymerase complexes that are functional yet resistant to rifampicin treatment [42,43]. Rifampicin targets RNA polymerase and is frequently used clinically to treat mycobacterial infections. RNA polymerases isolated from mistranslating strains show increased phenotypic resistance (or tolerance) to rifampicin, suggesting that mistranslated RNA polymerase carries amino acid replacements that decrease rifampicin binding [43]. Furthermore, introducing a high-fidelity mutation in the ribosomal protein RpsL (K43N) increases sensitivity to rifampicin. A more recent study from the Javid group shows that some clinical isolates of *Mycobacterium tuberculosis* contain mutations in GatCAB that decrease its stability, resulting in increased translational errors and rifampin tolerance [112]. These studies indicate that translational errors may provide benefits to cells through statistical translation of specific proteins.

### 3.4. Editing Defects in Host-Restricted Bacteria

*Mycoplasma* is a bacterial parasite that depends on a vertebrate host for survival and growth [113]. The genomes of *Mycoplasma* species are highly reduced, and several aaRSs either lack the entire editing domain or carry mutations at critical residues in the editing site [50]. Biochemical analyses confirm that *Mycoplasma* LeuRS and PheRS are indeed defective in editing [50,114]. This is consistent with mass spectrometry data revealing that Leu and Phe codons are mistranslated as Val and Tyr, respectively [50]. In addition to *Mycoplasma*, the majority of host-restricted bacteria (e.g., *Helicobacter*, *Borrelia*, and *Rickettsia*) have also lost the editing function in many aaRSs [51]. This is in sharp contrast to free-living bacteria that maintain robust editing activities. Whether statistical translation of the proteome provides benefits to the intracellular life cycle remains an open question.

### 3.5. Trans-Editing in Streptococci

Freestanding *trans*-editing factors provide another safeguard to hydrolyze misacylated tRNAs and enhance translational fidelity [9,10]. In *Streptococcus pneumoniae*, MurMN uses several tRNAs as substrates to synthesize peptides on the cell wall. It has been previously shown that MurM serves as a *trans*-editing factor to hydrolyze Ser-tRNA^Ala^ in vitro [115]. A recent study reveals that MurMN attenuates the stringent response and protects *S. pneumoniae* against acid stress [49]. Expressing the editing domain of AlaRS in the Δ*murMN* deletion strain partially suppresses the stringent response. It is thus hypothesized that the accumulation of misacylated tRNAs in the Δ*murMN* strain activates the stringent response, although it is unclear how this is achieved. Pneumococcal cells experience acidic pH in hosts [116]. Deleting *murMN* decreases macrophage phagocytosis via increased expression of an autolysin LytA [49]. Expressing the AlaRS editing domain in Δ*murMN* restores phagocytosis to the WT level, suggesting that accumulation of Ser-tRNA^Ala^ decreases macrophage phagocytosis. 

### 3.6. Aminoglycoside-Induced Biofilm Formation in Pseudomonas aeruginosa

Aminoglycoside antibiotics induce global translational errors and are lethal to Gram-positive and Gram-negative bacteria at high doses [75,117]. However, sublethal concentrations of tobramycin, an aminoglycoside antibiotic produced by *Streptomyces tenebrarius* and commonly used to treat *Pseudomonas aeruginosa*, have been shown to promote biofilm formation [36]. Other aminoglycosides tested show a similar stimulation on biofilm formation of *P. aeruginosa* and *E. coli*. This effect on biofilm formation depends on the aminoglycoside response regulator (Arr) gene. It is proposed that aminoglycosides, for an unknown mechanism, increase the phosphodiesterase activity of the Arr gene to inactivate c-di-GMP and promote biofilm formation [36].

### 3.7. Mistranslating ProRS/tRNA^Pro^ in Streptomyces

Variation or mutation in the tRNA sequence can lead to stop-codon suppression and missense errors [118,119,120]. A recent study reports that plant pathogens’ *Streptomyces* species encode a tRNA^ProA^ variant and an anomalous prolyl-tRNA synthetase isoform (ProRSx), which attaches Pro to tRNA^ProA^ and deliberately translates Ala codons as Pro [52]. In addition to ProRSx, *S. turgidiscabies* encodes two canonical ProRSs, which recognize the anticodon of normal tRNA^Pro^. The anticodon of tRNA^ProA^ is changed to AGC and recognizes GCU Ala codons. ProRSx has evolved to efficiently aminoacylate tRNA^ProA^ with Pro. Expressing the *S. turgidiscabies* ProRSx/tRNA^ProA^ pair leads to Pro misincorporation at Ala codons. The biological function of Pro mistranslation in *Streptomyces* remains an interesting open question. 

## 4. Concluding Remarks and Future Directions

In the past two decades, increasing numbers of studies have revealed remarkable plasticity and broad physiological roles of translational fidelity. In addition to genetic mutations, multiple environmental cues (e.g., stress conditions) affect various types of translational errors. Most of these studies are performed in laboratory conditions, and the errors are detected using reporters. Advances in high-sensitivity mass spectrometry technology would allow the detection and quantitation of different translational errors in bacterial and host proteomes under native conditions. It has been shown that certain types of translational errors benefit bacteria under stress conditions, yet the activation threshold of stress responses by mistranslation and the trade-off between benefits and harms remain to be determined. It is also puzzling why different types of translational errors sometimes induce distinct cellular responses and fitness changes. Our current understanding of how translational fidelity affects bacterial pathogens within hosts is spotty, and the underlying molecular mechanisms are largely unknown. Future studies are warranted to clarify the mechanisms and to investigate how different types of translational errors impact host interactions of various pathogens.

## Figures and Tables

**Table 1:** Translational errors in bacteria.

Error Types	Bacteria	Sources of Error	Phenotypes	Ref.
Global	*S. typhimurium*, *E. coli*	Mutations in *rpsD*	Decreased cell invasion and animal colonization; increased resistance against oxidative stress and heat; decreased motility	[26,27,28,29]
High-fidelity	*S. typhimurium*, *E. coli*	Mutations in *rpsL*	Decreased cell invasion and animal colonization; decreased resistance against oxidative stress; decreased motility	[26,27,28]
Readthrough	*E. coli*	Carbon starvation	Increased protein oxidation during aging	[30]
Thr → Ser	*E. coli*	Oxidative stress damages the editing site of ThrRS	Mild growth defect with excess Ser	[13,31,32]
Phe → *m*-Tyr	*E. coli*, *S. typhimurium*,	Oxidation of Tyr to *m*-Tyr	PheRS editing defect decreases growth under oxidative stress	[33,34]
Global	*E. coli*, *P. aeruginosa*	Aminoglycosides	Bactericidal; increased biofilm formation at sublethal doses	[35,36,37]
Initiation errors	*E. coli*	Deleting initiator tRNAs	Increased tolerance to fluoroquinolones and heat stress	[38]
Frameshift	*S. typhimurium*	Deleting *gidA* or *mnmE*	Mutations in *gidA* and *mnmE* decreases *Salmonella* invasion and host colonization	[39,40]
Readthrough	*E. coli*, *S. typhimurium*	Acid stress, excess sugar	May promote tolerance to acid stress	[15]
Readthrough	*E. coli*	Chloramphenicol, etc.	Unclear	[16]
Multiple AA → Met	*E. coli*	Anaerobic growth and antibiotic stress	Decreased MetRS succinylation increases Met misacylation	[41]
Gln → Glu, Asn → Asp	*M. smegmatis* *M. tuberculosis*	Mutations in tRNAs or *gatCAB*	Increased phenotypic resistance to rifampicin	[42,43]
Readthrough	*S. flexneri*	Deleting *miaA*	Decreased expression of virulence genes	[44]
Frameshift	ExPEC	Deleting or overexpressing *miaA*	Deleting *miaA* attenuates virulence	[45]
Ile → Val	*A. baylyi*	Editing-defective IleRS	Improved growth with excess Val	[46]
Ile → Val	*B. subtilis*	Editing-defective IleRS	Sporulation defect	[47]
Ala → Ser	*E. coli*	C666A mutation in AlaRS	Decreased motility	[48]
Ala → Ser	*S. pneumonia*	Deleting *murMN*	Decreased macrophage phagocytosis	[49]
Leu → Val, Phe → Tyr etc.	*M. mobile* and other host-restricted bacteria	Natural editing-defective aaRSs	May be adaptive to parasitic cycle	[50,51]
Ala → Pro	*Streptomyces* spp.	ProRS/tRNA^ProA^ pair	Unclear	[52]
Pro → Ala	*C. sticklandii*, *P. aeruginosa* etc.	Deleting *proX*	Unclear	[9,10]
Pro → Cys	*H. influenza*, *C. crescentus* etc.	Deleting *ybaK*	Unclear	[10,53]

## Data Availability

Not applicable.

## References

[1] Pontes M.H., Sevostyanova A., Groisman E.A. (2015). When Too Much ATP Is Bad for Protein Synthesis. J. Mol. Biol..

[2] Nissen P., Hansen J., Ban N., Moore P.B., Steitz T.A. (2000). The structural basis of ribosome activity in peptide bond synthesis. Science.

[3] Ogle J.M., Ramakrishnan V. (2005). Structural insights into translational fidelity. Annu. Rev. Biochem..

[4] Pape T., Wintermeyer W., Rodnina M. (1999). Induced fit in initial selection and proofreading of aminoacyl-tRNA on the ribosome. EMBO J..

[5] Rodnina M.V., Wintermeyer W. (2001). Ribosome fidelity: tRNA discrimination, proofreading and induced fit. Trends Biochem. Sci..

[6] Ibba M., Söll D. (2000). Aminoacyl-tRNA synthesis. Annu. Rev. Biochem..

[7] Ling J., Reynolds N., Ibba M. (2009). Aminoacyl-tRNA synthesis and translational quality control. Annu. Rev. Microbiol..

[8] Mascarenhas A.P., An S., Rosen A.E., Martinis S.A., Musier-Forsyth K., RajBhandary U.L., Köhrer C. (2008). Fidelity mechanisms of the aminoacyl-tRNA synthetases. Protein Engineering.

[9] Ahel I., Korencic D., Ibba M., Söll D. (2003). Trans-editing of mischarged tRNAs. Proc. Natl. Acad. Sci. USA.

[10] Vargas-Rodriguez O., Musier-Forsyth K. (2013). Exclusive use of trans-editing domains prevents proline mistranslation. J. Biol. Chem..

[11] Mohler K., Ibba M. (2017). Translational fidelity and mistranslation in the cellular response to stress. Nat. Microbiol..

[12] Evans C.R., Fan Y., Weiss K., Ling J. (2018). Errors during gene expression: Single-cell heterogeneity, stress resistance, and microbe-host interactions. mBio.

[13] Mohler K., Aerni H.R., Gassaway B., Ling J., Ibba M., Rinehart J. (2017). MS-READ: Quantitative measurement of amino acid incorporation. Biochim. Biophys. Acta.

[14] Wohlgemuth I., Garofalo R., Samatova E., Gunenc A.N., Lenz C., Urlaub H., Rodnina M.V. (2021). Translation error clusters induced by aminoglycoside antibiotics. Nat. Commun..

[15] Zhang H., Lyu Z., Fan Y., Evans C.R., Barber K.W., Banerjee K., Igoshin O.A., Rinehart J., Ling J. (2020). Metabolic stress promotes stop-codon readthrough and phenotypic heterogeneity. Proc. Natl. Acad. Sci. USA.

[16] Fan Y., Evans C.R., Barber K.W., Banerjee K., Weiss K.J., Margolin W., Igoshin O.A., Rinehart J., Ling J. (2017). Heterogeneity of stop codon readthrough in single bacterial cells and implications for population fitness. Mol. Cell.

[17] Dunn J.G., Foo C.K., Belletier N.G., Gavis E.R., Weissman J.S. (2013). Ribosome profiling reveals pervasive and regulated stop codon readthrough in *Drosophila melanogaster*. eLife.

[18] Wangen J.R., Green R. (2020). Stop codon context influences genome-wide stimulation of termination codon readthrough by aminoglycosides. eLife.

[19] Kramer E.B., Vallabhaneni H., Mayer L.M., Farabaugh P.J. (2010). A comprehensive analysis of translational missense errors in the yeast *Saccharomyces cerevisiae*. RNA.

[20] Kramer E.B., Farabaugh P.J. (2007). The frequency of translational misreading errors in *E. coli* is largely determined by tRNA competition. RNA.

[21] Battesti A., Majdalani N., Gottesman S. (2011). The RpoS-mediated general stress response in *Escherichia coli*. Annu. Rev. Microbiol..

[22] Storz G., Imlay J.A. (1999). Oxidative stress. Curr. Opin. Microbiol..

[23] Fang F.C., Frawley E.R., Tapscott T., Vazquez-Torres A. (2016). Bacterial stress responses during host infection. Cell Host Microbe.

[24] Hampton M.B., Kettle A.J., Winterbourn C.C. (1998). Inside the neutrophil phagosome: Oxidants, myeloperoxidase, and bacterial killing. Blood.

[25] Winterbourn C.C., Hampton M.B., Livesey J.H., Kettle A.J. (2006). Modeling the reactions of superoxide and myeloperoxidase in the neutrophil phagosome: Implications for microbial killing. J. Biol. Chem..

[26] Fan Y., Thompson L., Lyu Z., Cameron T.A., De Lay N.R., Krachler A.M., Ling J. (2019). Optimal translational fidelity is critical for *Salmonella* virulence and host interactions. Nucleic Acids Res..

[27] Bjorkman J., Samuelsson P., Andersson D.I., Hughes D. (1999). Novel ribosomal mutations affecting translational accuracy, antibiotic resistance and virulence of *Salmonella* Typhimurium. Mol. Microbiol..

[28] Fan Y., Wu J., Ung M.H., De Lay N., Cheng C., Ling J. (2015). Protein mistranslation protects bacteria against oxidative stress. Nucleic Acids Res..

[29] Evans C.R., Fan Y., Ling J. (2019). Increased mistranslation protects *E. coli* from protein misfolding stress due to activation of a RpoS-dependent heat shock response. FEBS Lett..

[30] Ballesteros M., Fredriksson A., Henriksson J., Nystrom T. (2001). Bacterial senescence: Protein oxidation in non-proliferating cells is dictated by the accuracy of the ribosomes. EMBO J..

[31] Ling J., Söll D. (2010). Severe oxidative stress induces protein mistranslation through impairment of an aminoacyl-tRNA synthetase editing site. Proc. Natl. Acad. Sci. USA.

[32] Wu J., Fan Y., Ling J. (2014). Mechanism of oxidant-induced mistranslation by threonyl-tRNA synthetase. Nucleic Acids Res..

[33] Bullwinkle T., Reynolds N.M., Raina M., Moghal A.B., Matsa E., Rajkovic A., Kayadibi H., Fazlollahi F., Ryan C., Howitz N. (2014). Oxidation of cellular amino acid pools leads to cytotoxic mistranslation of the genetic code. eLife.

[34] Steiner R.E., Kyle A.M., Ibba M. (2019). Oxidation of phenylalanyl-tRNA synthetase positively regulates translational quality control. Proc. Natl. Acad. Sci. USA.

[35] Davies J., Gorini L., Davis B.D. (1965). Misreading of RNA codewords induced by aminoglycoside antibiotics. Mol. Pharmacol..

[36] Hoffman L.R., D’Argenio D.A., MacCoss M.J., Zhang Z., Jones R.A., Miller S.I. (2005). Aminoglycoside antibiotics induce bacterial biofilm formation. Nature.

[37] Kohanski M.A., Dwyer D.J., Wierzbowski J., Cottarel G., Collins J.J. (2008). Mistranslation of membrane proteins and two-component system activation trigger antibiotic-mediated cell death. Cell.

[38] Samhita L., Raval P.K., Agashe D. (2020). Global mistranslation increases cell survival under stress in Escherichia coli. PLoS Genet..

[39] Bregeon D., Colot V., Radman M., Taddei F. (2001). Translational misreading: A tRNA modification counteracts a +2 ribosomal frameshift. Genes Dev..

[40] Shippy D.C., Eakley N.M., Lauhon C.T., Bochsler P.N., Fadl A.A. (2013). Virulence characteristics of *Salmonella* following deletion of genes encoding the tRNA modification enzymes GidA and MnmE. Microb. Pathog..

[41] Schwartz M.H., Waldbauer J.R., Zhang L., Pan T. (2016). Global tRNA misacylation induced by anaerobiosis and antibiotic exposure broadly increases stress resistance in *Escherichia coli*. Nucleic Acids Res..

[42] Su H.W., Zhu J.H., Li H., Cai R.J., Ealand C., Wang X., Chen Y.X., Kayani M.U., Zhu T.F., Moradigaravand D. (2016). The essential mycobacterial amidotransferase GatCAB is a modulator of specific translational fidelity. Nat. Microbiol..

[43] Javid B., Sorrentino F., Toosky M., Zheng W., Pinkham J.T., Jain N., Pan M., Deighan P., Rubin E.J. (2014). Mycobacterial mistranslation is necessary and sufficient for rifampicin phenotypic resistance. Proc. Natl. Acad. Sci. USA.

[44] Durand J.M., Dagberg B., Uhlin B.E., Bjork G.R. (2000). Transfer RNA modification, temperature and DNA superhelicity have a common target in the regulatory network of the virulence of *Shigella flexneri*: The expression of the virF gene. Mol. Microbiol..

[45] Fleming B.A., Blango M.G., Rousek A.A., Kincannon W.M., Tran A., Lewis A.J., Russell C.W., Zhou Q., Baird L.M., Barber A.E. (2022). A tRNA modifying enzyme as a tunable regulatory nexus for bacterial stress responses and virulence. Nucleic Acids Res..

[46] Bacher J.M., Waas W.F., Metzgar D., Crécy-Lagard V., Schimmel P. (2007). Genetic code ambiguity confers a selective advantage on *Acinetobacter baylyi*. J. Bacteriol..

[47] Kermgard E., Yang Z., Michel A.M., Simari R., Wong J., Ibba M., Lazazzera B.A. (2017). Quality control by isoleucyl-tRNA synthetase of *Bacillus subtilis* is required for efficient sporulation. Sci. Rep..

[48] Kelly P., Backes N., Mohler K., Buser C., Kavoor A., Rinehart J., Phillips G., Ibba M. (2019). Alanyl-tRNA synthetase quality control prevents global dysregulation of the *Escherichia coli* proteome. mBio.

[49] Aggarwal S.D., Lloyd A.J., Yerneni S.S., Narciso A.R., Shepherd J., Roper D.I., Dowson C.G., Filipe S.R., Hiller N.L. (2021). A molecular link between cell wall biosynthesis, translation fidelity, and stringent response in Streptococcus pneumoniae. Proc. Natl. Acad. Sci. USA.

[50] Li L., Boniecki M.T., Jaffe J.D., Imai B.S., Yau P.M., Luthey-Schulten Z.A., Martinis S.A. (2011). Naturally occurring aminoacyl-tRNA synthetases editing-domain mutations that cause mistranslation in *Mycoplasma* parasites. Proc. Natl. Acad. Sci. USA.

[51] Melnikov S.V., van den Elzen A., Stevens D.L., Thoreen C.C., Soll D. (2018). Loss of protein synthesis quality control in host-restricted organisms. Proc. Natl. Acad. Sci. USA.

[52] Vargas-Rodriguez O., Badran A.H., Hoffman K.S., Chen M., Crnkovic A., Ding Y., Krieger J.R., Westhof E., Soll D., Melnikov S. (2021). Bacterial translation machinery for deliberate mistranslation of the genetic code. Proc. Natl. Acad. Sci. USA.

[53] An S., Musier-Forsyth K. (2004). Trans-editing of Cys-tRNA^Pro^ by *Haemophilus influenzae* YbaK protein. J. Biol. Chem..

[54] Imlay J.A. (2008). Cellular defenses against superoxide and hydrogen peroxide. Annu. Rev. Biochem..

[55] Imlay J.A. (2013). The molecular mechanisms and physiological consequences of oxidative stress: Lessons from a model bacterium. Nat. Rev. Microbiol..

[56] Dock-Bregeon A., Sankaranarayanan R., Romby P., Caillet J., Springer M., Rees B., Francklyn C.S., Ehresmann C., Moras D. (2000). Transfer RNA-mediated editing in threonyl-tRNA synthetase. The class II solution to the double discrimination problem. Cell.

[57] Beebe K., Ribas de Pouplana L., Schimmel P. (2003). Elucidation of tRNA-dependent editing by a class II tRNA synthetase and significance for cell viability. EMBO J..

[58] Dock-Bregeon A.C., Rees B., Torres-Larios A., Bey G., Caillet J., Moras D. (2004). Achieving error-free translation; the mechanism of proofreading of threonyl-tRNA synthetase at atomic resolution. Mol. Cell..

[59] Zhang H., Wu J., Lyu Z., Ling J. (2021). Impact of alanyl-tRNA synthetase editing deficiency in yeast. Nucleic Acids Res..

[60] Kavoor A., Kelly P., Ibba M. (2022). *Escherichia coli* alanyl-tRNA synthetase maintains proofreading activity and translational accuracy under oxidative stress. J. Biol. Chem..

[61] Ling J., Roy H., Ibba M. (2007). Mechanism of tRNA-dependent editing in translational quality control. Proc. Natl. Acad. Sci. USA.

[62] Roy H., Ling J., Irnov M., Ibba M. (2004). Post-transfer editing in vitro and in vivo by the beta subunit of phenylalanyl-tRNA synthetase. EMBO J..

[63] Ling J., Yadavalli S.S., Ibba M. (2007). Phenylalanyl-tRNA synthetase editing defects result in efficient mistranslation of phenylalanine codons as tyrosine. RNA.

[64] Bullwinkle T., Lazazzera B., Ibba M. (2014). Quality control and infiltration of translation by amino acids outside of the genetic code. Annu. Rev. Genet..

[65] Srinivas P., Steiner R.E., Pavelich I.J., Guerrero-Ferreira R., Juneja P., Ibba M., Dunham C.M. (2021). Oxidation alters the architecture of the phenylalanyl-tRNA synthetase editing domain to confer hyperaccuracy. Nucleic Acids Res..

[66] Willi J., Kupfer P., Evequoz D., Fernandez G., Katz A., Leumann C., Polacek N. (2018). Oxidative stress damages rRNA inside the ribosome and differentially affects the catalytic center. Nucleic Acids Res..

[67] Shcherbik N., Pestov D.G. (2019). The Impact of Oxidative Stress on Ribosomes: From Injury to Regulation. Cells.

[68] Kyuma T., Kizaki H., Ryuno H., Sekimizu K., Kaito C. (2015). 16S rRNA methyltransferase KsgA contributes to oxidative stress resistance and virulence in *Staphylococcus aureus*. Biochimie.

[69] Youngman E.M., McDonald M.E., Green R. (2008). Peptide release on the ribosome: Mechanism and implications for translational control. Annu. Rev. Microbiol..

[70] Kuhlenkoetter S., Wintermeyer W., Rodnina M.V. (2011). Different substrate-dependent transition states in the active site of the ribosome. Nature.

[71] Shaw J.J., Trobro S., He S.L., Aqvist J., Green R. (2012). A Role for the 2’ OH of peptidyl-tRNA substrate in peptide release on the ribosome revealed through RF-mediated rescue. Chem. Biol..

[72] Indrisiunaite G., Pavlov M.Y., Heurgue-Hamard V., Ehrenberg M. (2015). On the pH dependence of class-1 RF-dependent termination of mRNA translation. J. Mol. Biol..

[73] Lyu Z., Villanueva P., O’Malley L., Murphy P., Ling J. (2022). Genome-wide screening reveals metabolic regulation of translational fidelity. BioRxiv.

[74] Zheng D., Constantinidou C., Hobman J.L., Minchin S.D. (2004). Identification of the CRP regulon using in vitro and in vivo transcriptional profiling. Nucleic Acids Res..

[75] Becker B., Cooper M.A. (2013). Aminoglycoside antibiotics in the 21st century. ACS Chem. Biol..

[76] Carter A.P., Clemons W.M., Brodersen D.E., Morgan-Warren R.J., Wimberly B.T., Ramakrishnan V. (2000). Functional insights from the structure of the 30S ribosomal subunit and its interactions with antibiotics. Nature.

[77] Demirci H., Murphy F.t., Murphy E., Gregory S.T., Dahlberg A.E., Jogl G. (2013). A structural basis for streptomycin-induced misreading of the genetic code. Nat. Commun..

[78] Davis B.D., Chen L.L., Tai P.C. (1986). Misread protein creates membrane channels: An essential step in the bactericidal action of aminoglycosides. Proc. Natl. Acad. Sci. USA.

[79] Fredriksson A., Ballesteros M., Peterson C.N., Persson O., Silhavy T.J., Nystrom T. (2007). Decline in ribosomal fidelity contributes to the accumulation and stabilization of the master stress response regulator sigmaS upon carbon starvation. Genes Dev..

[80] Davies J., Gilbert W., Gorini L. (1964). Streptomycin, suppression, and the code. Proc. Natl. Acad. Sci. USA.

[81] Agarwal D., Kamath D., Gregory S.T., O’Connor M. (2015). Modulation of decoding fidelity by ribosomal proteins S4 and S5. J. Bacteriol..

[82] Lyu Z., Ling J. (2022). Increase in ribosomal fidelity benefits *Salmonella* upon bile salt exposure. Genes.

[83] El Yacoubi B., Bailly M., de Crecy-Lagard V. (2012). Biosynthesis and function of posttranscriptional modifications of transfer RNAs. Annu. Rev. Genet..

[84] Suzuki T. (2021). The expanding world of tRNA modifications and their disease relevance. Nat. Rev. Mol. Cell Biol..

[85] Petrullo L.A., Gallagher P.J., Elseviers D. (1983). The role of 2-methylthio-N6-isopentenyladenosine in readthrough and suppression of nonsense codons in *Escherichia coli*. Mol. Gen. Genet..

[86] Vacher J., Grosjean H., Houssier C., Buckingham R.H. (1984). The effect of point mutations affecting *Escherichia coli* tryptophan tRNA on anticodon-anticodon interactions and on UGA suppression. J. Mol. Biol..

[87] Ericson J.U., Bjork G.R. (1986). Pleiotropic effects induced by modification deficiency next to the anticodon of tRNA from *Salmonella* Typhimurium LT2. J. Bacteriol..

[88] Blum P.H. (1988). Reduced leu operon expression in a miaA mutant of Salmonella typhimurium. J. Bacteriol..

[89] Gamper H.B., Masuda I., Frenkel-Morgenstern M., Hou Y.M. (2015). Maintenance of protein synthesis reading frame by EF-P and m(1)G37-tRNA. Nat. Commun..

[90] Bystrom A.S., Bjork G.R. (1982). The structural gene (trmD) for the tRNA(m1G)methyltransferase is part of a four polypeptide operon in *Escherichia coli* K-12. Mol. Gen. Genet..

[91] Bjork G.R., Wikstrom P.M., Bystrom A.S. (1989). Prevention of translational frameshifting by the modified nucleoside 1-methylguanosine. Science.

[92] Gamper H., Li H., Masuda I., Miklos Robkis D., Christian T., Conn A.B., Blaha G., Petersson E.J., Gonzalez R.L., Hou Y.M. (2021). Insights into genome recoding from the mechanism of a classic +1-frameshifting tRNA. Nat. Commun..

[93] Masuda I., Hwang J.Y., Christian T., Maharjan S., Mohammad F., Gamper H., Buskirk A.R., Hou Y.M. (2021). Loss of N(1)-methylation of G37 in tRNA induces ribosome stalling and reprograms gene expression. eLife.

[94] Hou Y.M., Matsubara R., Takase R., Masuda I., Sulkowska J.I. (2017). TrmD: A Methyl Transferase for tRNA Methylation With m(1)G37. Enzymes.

[95] Kobayashi K., Ehrlich S.D., Albertini A., Amati G., Andersen K.K., Arnaud M., Asai K., Ashikaga S., Aymerich S., Bessieres P. (2003). Essential *Bacillus subtilis* genes. Proc. Natl. Acad. Sci. USA.

[96] O’Dwyer K., Watts J.M., Biswas S., Ambrad J., Barber M., Brule H., Petit C., Holmes D.J., Zalacain M., Holmes W.M. (2004). Characterization of *Streptococcus pneumoniae* TrmD, a tRNA methyltransferase essential for growth. J. Bacteriol..

[97] Bjork G.R., Jacobsson K., Nilsson K., Johansson M.J., Bystrom A.S., Persson O.P. (2001). A primordial tRNA modification required for the evolution of life?. EMBO J..

[98] Masuda I., Matsubara R., Christian T., Rojas E.R., Yadavalli S.S., Zhang L., Goulian M., Foster L.J., Huang K.C., Hou Y.M. (2019). tRNA methylation is a global determinant of bacterial multi-drug resistance. Cell Syst..

[99] Groisman E.A., Hollands K., Kriner M.A., Lee E.J., Park S.Y., Pontes M.H. (2013). Bacterial Mg^2+^ homeostasis, transport, and virulence. Annu. Rev. Genet..

[100] Gall A.R., Datsenko K.A., Figueroa-Bossi N., Bossi L., Masuda I., Hou Y.M., Csonka L.N. (2016). Mg^2+^ regulates transcription of *mgtA* in *Salmonella* Typhimurium via translation of proline codons during synthesis of the MgtL peptide. Proc. Natl. Acad. Sci. USA.

[101] Yamada Y., Murao K., Ishikura H. (1981). 5-(carboxymethylaminomethyl)-2-thiouridine, a new modified nucleoside found at the first letter position of the anticodon. Nucleic Acids Res..

[102] Meyer S., Wittinghofer A., Versees W. (2009). G-domain dimerization orchestrates the tRNA wobble modification reaction in the MnmE/GidA complex. J. Mol. Biol..

[103] Yim L., Moukadiri I., Bjork G.R., Armengod M.E. (2006). Further insights into the tRNA modification process controlled by proteins MnmE and GidA of *Escherichia coli*. Nucleic Acids Res..

[104] Urbonavicius J., Qian Q., Durand J.M., Hagervall T.G., Bjork G.R. (2001). Improvement of reading frame maintenance is a common function for several tRNA modifications. EMBO J..

[105] Urbonavicius J., Stahl G., Durand J.M., Ben Salem S.N., Qian Q., Farabaugh P.J., Bjork G.R. (2003). Transfer RNA modifications that alter +1 frameshifting in general fail to affect -1 frameshifting. RNA.

[106] Jager G., Nilsson K., Bjork G.R. (2013). The phenotype of many independently isolated +1 frameshift suppressor mutants supports a pivotal role of the P-site in reading frame maintenance. PLoS ONE.

[107] Shippy D.C., Heintz J.A., Albrecht R.M., Eakley N.M., Chopra A.K., Fadl A.A. (2012). Deletion of glucose-inhibited division (*gidA*) gene alters the morphological and replication characteristics of Salmonella enterica Serovar typhimurium. Arch. Microbiol..

[108] Okamoto S., Tamaru A., Nakajima C., Nishimura K., Tanaka Y., Tokuyama S., Suzuki Y., Ochi K. (2007). Loss of a conserved 7-methylguanosine modification in 16S rRNA confers low-level streptomycin resistance in bacteria. Mol. Microbiol..

[109] Mikheil D.M., Shippy D.C., Eakley N.M., Okwumabua O.E., Fadl A.A. (2012). Deletion of gene encoding methyltransferase (*gidB*) confers high-level antimicrobial resistance in *Salmonella*. J. Antibiot..

[110] Sheppard K., Akochy P.M., Salazar J.C., Söll D. (2007). The *Helicobacter pylori* amidotransferase GatCAB is equally efficient in glutamine-dependent transamidation of Asp-tRNA^Asn^ and Glu-tRNA^Gln^. J. Biol. Chem..

[111] Nakamura A., Yao M., Chimnaronk S., Sakai N., Tanaka I. (2006). Ammonia channel couples glutaminase with transamidase reactions in GatCAB. Science.

[112] Li Y.Y., Cai R.J., Yang J.Y., Hendrickson T.L., Xiang Y., Javid B. (2021). Clinically relevant mutations of mycobacterial GatCAB inform regulation of translational fidelity. mBio.

[113] Rottem S. (2003). Interaction of mycoplasmas with host cells. Physiol. Rev..

[114] Han N.C., Kavoor A., Ibba M. (2022). Characterizing the amino acid activation center of the naturally editing-deficient aminoacyl-tRNA synthetase PheRS in *Mycoplasma mobile*. FEBS Lett..

[115] Shepherd J., Ibba M. (2013). Lipid II-independent trans editing of mischarged tRNAs by the penicillin resistance factor MurM. J. Biol. Chem..

[116] Park S.Y., Kim I.S. (2013). Identification of macrophage genes responsive to extracellular acidification. Inflamm. Res.

[117] Aguirre Rivera J., Larsson J., Volkov I.L., Seefeldt A.C., Sanyal S., Johansson M. (2021). Real-time measurements of aminoglycoside effects on protein synthesis in live cells. Proc. Natl. Acad. Sci. USA.

[118] Eggertsson G., Söll D. (1988). Transfer ribonucleic acid-mediated suppression of termination codons in *Escherichia coli*. Microbiol. Rev..

[119] Santos M.A., Tuite M.F. (1995). The CUG codon is decoded in vivo as serine and not leucine in *Candida albicans*. Nucleic Acids Res..

[120] Lant J.T., Berg M.D., Heinemann I.U., Brandl C.J., O’Donoghue P. (2019). Pathways to disease from natural variations in human cytoplasmic tRNAs. J. Biol. Chem..

